# BL46XU: an applied hard X-ray photoelectron spectroscopy beamline HAXPES II at SPring-8

**DOI:** 10.1107/S1600577525007660

**Published:** 2025-10-28

**Authors:** Satoshi Yasuno, Yasumasa Takagi, Akira Yasui, Okkyun Seo, Taito Osaka, Michihiro Sugahara, Yasunori Senba, Hiroshi Yamazaki, Takahisa Koyama, Satsuki Shimizu, Takamitsu Saito, Haruhiko Ohashi, Naomi Kawamura, Kyo Nakajima, Koji Motomura, Tappei Nishihara, Masugu Sato, Yusuke Tamenori, Makina Yabashi

**Affiliations:** a Japan Synchrotron Radiation Research Institute 1-1-1 Kouto Sayo Hyogo679-5198 Japan; b RIKEN SPring-8 Center, 1-1-1 Kouto, Sayo, Hyogo679-5148, Japan; University of Essex, United Kingdom

**Keywords:** hard X-ray photoelectron spectroscopy, ambient-pressure HAXPES, beamlines, high throughput

## Abstract

The BL46XU beamline of SPring-8 has been reorganized into a beamline dedicated to hard X-ray photoelectron spectroscopy (HAXPES) to meet the increasing demand for various HAXPES based measurements. Two specialized HAXPES instruments, namely, (i) a high-throughput HAXPES system specialized for automated measurements and (ii) an ambient pressure HAXPES system with a focus on measurements under a gas atmosphere, provide advanced capabilities for characterizing bulk-sensitive electronic and chemical states in a variety of research fields. To enhance the capabilities further, several X-ray optical instruments have been introduced. Two types of double channel-cut monochromators [Si(220) and Si(311)] have been installed in the optics hutch, allowing users to select the optimum energy resolution and flux in a wide photon-energy range (4.9–21.8 keV) while keeping a fixed-exit condition. In addition, a focusing mirror to provide a high-ﬂux microbeam has been arranged for each HAXPES system. In this article, the design and performance of the beamline as well as some recent scientific results are outlined.

## Abstract 2

The BL46XU beamline at SPring-8 has been restructured for hard X-ray photoelectron spectroscopy (HAXPES), featuring two specialized HAXPES instruments and advanced X-ray optics for high-throughput and ambient-pressure measurements. This article outlines the beamline design, performance and recent scientific achievements enabled by these upgrades.

## 1. Introduction

Owing to the development of third-generation high-brilliance synchrotron radiation sources and relevant instruments, hard X-ray photoelectron spectroscopy (HAXPES) has recently become a powerful tool for investigating the chemical states and electronic structures of various materials. The most advantageous feature of HAXPES is its capability to probe bulk and buried interfaces lying at depths of several tens of nanometres due to its large probing depth, which are inaccessible in conventional photoelectron spectroscopy with vacuum ultraviolet or soft X-ray beams (Tanuma *et al.*, 2003 ▸; Kobayashi, 2009 ▸). Therefore, HAXPES has been utilized for versatile analysis in various research fields, such as electronic devices, inorganic and organic materials, and rechargeable batteries (Kobayashi *et al.*, 2003 ▸; Yabuuchi *et al.*, 2011 ▸; Watanabe *et al.*, 2016 ▸). At present, HAXPES has been installed in various beamlines of synchrotron radiation facilities worldwide (Kalha *et al.*, 2021 ▸). At SPring-8, HAXPES had been operated on several beamlines including public beamlines BL09XU, BL47XU (Ikenaga *et al.*, 2013 ▸; Ikenaga *et al.*, 2018 ▸) and BL46XU (Yasuno *et al.*, 2016 ▸), while the operation and technical development has been carried out independently. Recently, we have consolidated major HAXPES activities at SPring-8 into BL09XU and BL46XU with major revisions of beamline configurations and instruments. BL09XU (HAXPES I) is mainly organized for advanced HAXPES measurements, such as resonant analysis and three-dimensional microanalysis (Yasui *et al.*, 2023 ▸), while BL46XU (HAXPES II) has been upgraded for high-throughput analysis and measurements in ambient-pressure environments.

In this article, we present the latest design of the upgraded beamline BL46XU and report test results that verify its capabilities.

## 2. Beamline overview

### 2.1. Design

**Figure 1:**
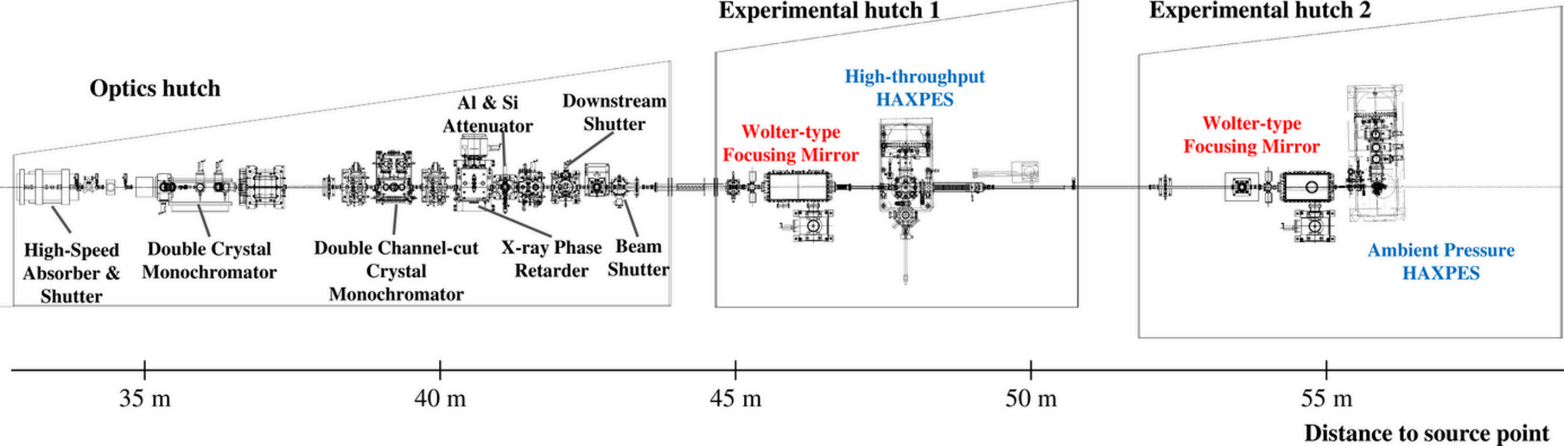
Beamline layout of BL46XU. All optical components except the focusing system are located in the OH. Two HAXPES systems with different specifications of photoelectron analyzers and Wolter-type focusing mirrors are installed in EH1 and EH2, respectively.

BL46XU is equipped with a 4.5 m in-vacuum linear undulator with a period length of 32 mm (Hara *et al.*, 1998 ▸; Kitamura, 1998 ▸). The beam from the undulator is shaped with a primary slit located at the front-end section (FE slit) to reduce the heat loads on the optical components downstream. The beamline layout is shown in Fig. 1 ▸. In the optics hutch (OH), a liquid-nitro­gen-cooled double-crystal monochromator with Si(111) crystal pairs is utilized to deliver the monochromatic beam to the experimental hutch (EH) while keeping a fixed-exit condition. Two types of double-crystal channel-cut monochromators (DCCMs) are installed to function as high-resolution monochromators (HRMs) for reducing the energy bandwidth of the X-rays. Note that a downstream shutter is located at the end of the OH in order to keep the heat loads to the HRMs constant even when the EH is open. Two HAXPES instruments with different speciﬁcations are installed in each of the EHs. To form microbeams at the sample positions, two Wolter-type X-ray mirrors are installed in both EH1 and EH2. The focusing mirror of EH1 can be easily retracted from the optical axis for transporting the X-ray beam to EH2 so that one can switch EH to be used with a short time. Details of the optical components and HAXPES systems are provided in the following subsections.

### 2.2. Optical components

As an HRM, a single channel-cut crystal monochromator (CCM) with Si(333) was originally utilized in BL46XU with limited photon energies of 6 keV, 8 keV and 10 keV. With the present upgrade, we installed two types of DCCMs to extend the photon energy range while keeping a fixed-exit condition, as the beam-height deviation given by the ﬁrst CCM is compensated by the second CCM. We prepared two sets of the channel-cut monochromator crystals of Si(220) and Si(311). The low-order reﬂections of Si(220)/(311) provide a high-intensity beam with a moderate resolution that is suitable for HAXPES measurement over the wide energy range 4.6–21.8 keV while changing the Bragg angle from 10° to 45°. They can be easily switched by horizontal translations. The parameters are summarized in Table 1 ▸. In practice, however, due to the limited undulator gap range, the minimum accessible photon energy is restricted to above 4.9 keV. On the other hand, the maximum photon energy is 21.8 keV, which is determined by the Bragg angle of the Si(311) DCCM. As described later, photon energies above 12 keV are utilized without mirror focusing due to the cut-off energy limitation of the Wolter-type focusing mirror. The design of an X-ray phase retarder system is based on that of the system installed at BL09XU of SPring-8 (Yasui *et al.*, 2023 ▸). Diamond (001) crystals with thicknesses of 0.5 mm are operated in the 220 Laue geometry while covering the energy range 4.9–12 keV. The diamond crystals have a strain-relief structure and are mechanically mounted on dedicated holders with ultra-high-vacuum compatibility.

Two monolithic Wolter-type focusing mirrors, which have an elliptical and a hyperbolic surface shape on a single substrate, were adopted for the focusing system of EH1 and EH2 (Senba *et al.*, 2020 ▸). The mirrors are installed in an ultra-high-vacuum chamber to reduce unwanted contamination on the surface. The focused beam sizes are designed to 0.6 µm (V) × 23 µm (H) and 0.3 µm (V) × 13 µm (H) for EH1 and EH2, respectively, when the FE slit is fully opened. Here, note that this Wolter mirror is designed to directly focus the X-ray source, and the focused beam size is determined by the source size multiplied by the magnification factor. Therefore, the larger horizontal beam size compared with the vertical one is attributed to the source size. Reducing the horizontal aperture size of the FE slit has little effect on the focal spot size. This mirror has a high angular tolerance of about ±400 µrad to the pitching error, which simplifies the refocusing procedure when switching the EH and helps it maintain a good stability. The optical parameters are summarized in Table 2 ▸.

### 2.3. HAXPES systems

**Figure 2:**
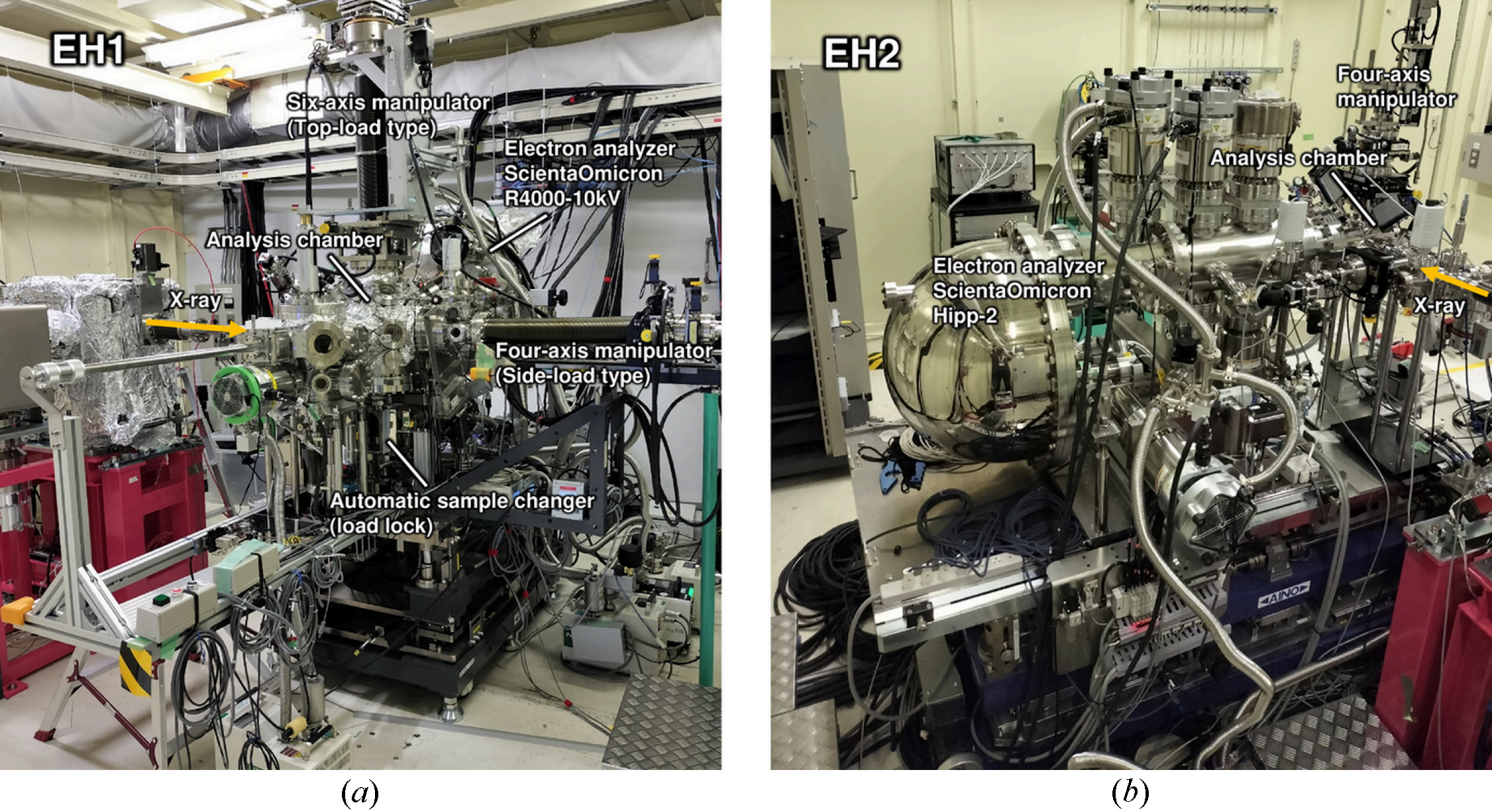
Photograph of HAXPES systems installed in (*a*) EH1 and (*b*) EH2. Focusing mirrors with different speciﬁcations are installed in EH1 and EH2, respectively.

Two photoelectron analyzers (Scienta Omicron AB), the R4000-10 kV and Hipp-2, are installed in EH1 and EH2, respectively, as shown in Fig. 2 ▸. Both analyzers can detect high-kinetic-energy photoelectrons up to 10 keV, allowing us to analyze electronic states in deeper buried interfaces compared with conventional soft X-ray photoelectron spectroscopy. A high-throughput HAXPES system specialized for automated measurements was installed in the upstream EH1. This system is based on the general-purpose HAXPES system previously installed at BL46XU EH2 (Yasuno *et al.*, 2016 ▸) and relocated to EH1. In addition, we have recently developed a high-energy HAXPES (HE-HAXPES) technique excited by photon energies up to 30 keV using a side-load manipulator to increase the probing depth (Yasuno *et al.*, 2023 ▸). The ambient-pressure HAXPES system in EH2 is capable of measurements under a gas atmosphere. Note that this system was previously operated at BL36XU at SPring-8 (Takagi *et al.*, 2014 ▸), primarily for fuel cell analysis (Takagi, Wang *et al.*, 2017 ▸; Yu *et al.*, 2017 ▸). The system is now available to general users for a broader range of applications, including solid–gas reactions, photocatalysis and electrochemical reactions. Here, note that we have evaluated the transmission function of the R4000-10 kV analyzer installed in EH1 using an experimental method (Berresheim *et al.*, 1991 ▸). We confirmed that the transmission function varies as a function of electron kinetic energy, consistent with previously reported trends observed for the same type of photoelectron analyzer (Weiland *et al.*, 2013 ▸; Drera *et al.*, 2014 ▸). The transmission function is crucial for quantitative analysis, especially when applying empirical relative sensitivity factors to other photoelectron analyzers (Yasuno *et al.*, 2020 ▸).

### 2.4. Control system

There has been an increasing need to perform highly efficient or advanced measurements by linking and controlling multiple instruments, such as an automated measurement system with a sample exchanger, X-ray energy-dependent measurement and various *in situ* measurements. Therefore, the instrument control system has also been updated. All the optical instruments, sample manipulators and automated sample exchangers can be controlled by the BL-774 instrument control platform (Nakajima *et al.*, 2022 ▸). This is a Python based system that allows for the control of different types of instruments, such as motor-driven devices, a current amplifier and a counter in a uniﬁed manner. Device control and communication management can be carried out by web based software.

## 3. Performance

### 3.1. Energy resolution

**Figure 3:**
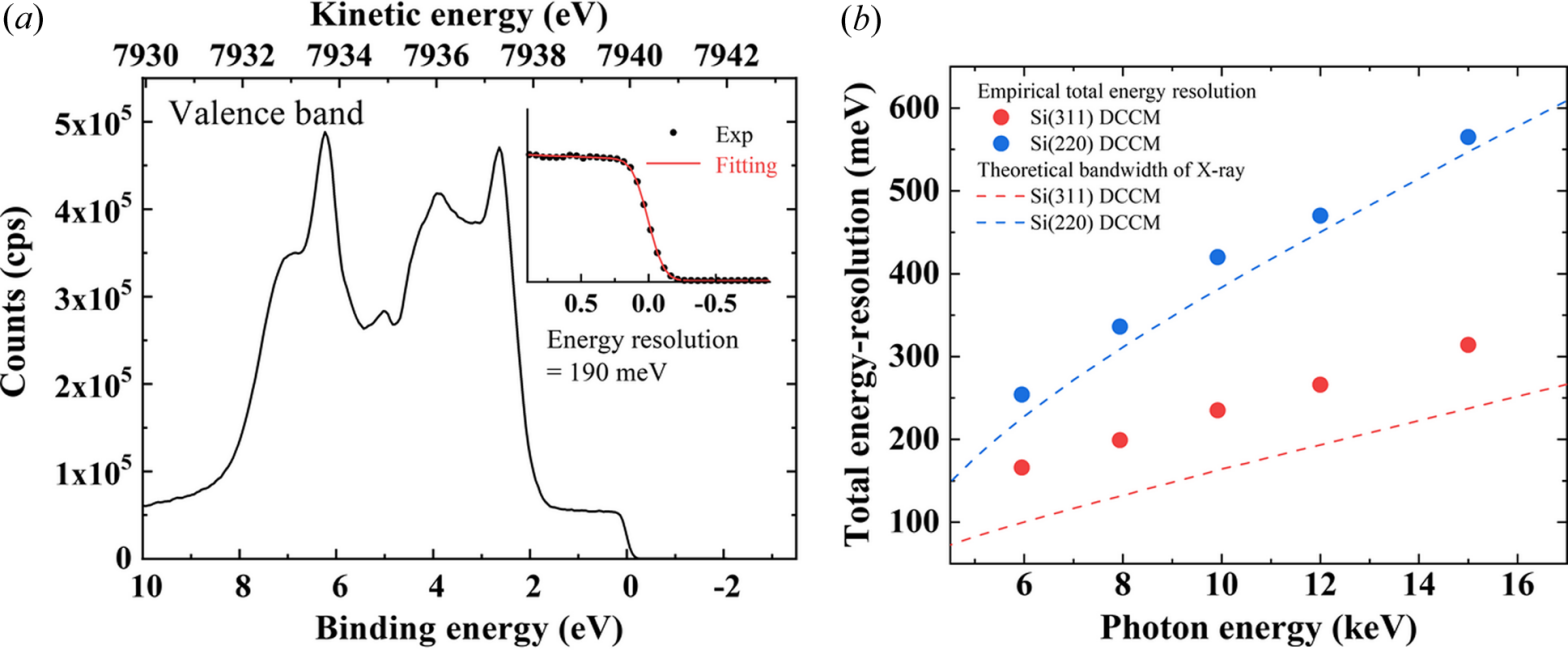
(*a*) Valence band spectrum of an Au plate measured using an Si(311) DCCM at a photon energy of 7.94 keV. The inset depicts a magnified view of the Fermi edge region. Experimental spectrum (filled black circles) is fitted with the Fermi–Dirac distribution function (solid red line). (*b*) Total energy resolution as a function of the photon energy and index of the DCCM crystal. The solid circles indicate those obtained by Fermi-edge measurements of an Au sample and the dashed lines are the theoretical energy bandwidths of the incident X-rays after DCCMs.

To evaluate the total energy resolution (Δ*E*_T_) dependence of X-ray energy, the valence band spectra of the Au plate sample were collected at X-ray energies from 5.95 keV to 15.0 keV with Si(220) or Si(311) DCCMs using the high-throughput HAXPES system in EH1 with an analyzer pass-energy, *E*_p_, of 100 eV, at room temperature. Note that the photoelectrons emerging from the sample applied bias positively for the measurements with X-ray energyies of 12 keV and 15 keV to decrease the photoelectron energy below 10 keV and to enable measurement with the analyzer (Yasuno *et al.*, 2023 ▸). As a typical example, the result of the X-ray energy of 7.94 keV with Si(311) DCCMs is shown in Fig. 3 ▸(*a*). The Fermi edge and detailed valence band structures are clearly observed in the spectrum. Δ*E*_T_ including the bandwidth of the incident X-ray is determined to be 190 meV by fitting the Fermi–Dirac distribution function [solid red line in the inset of Fig. 3 ▸(*a*)], which is sufficient for analyzing the chemical and electronic states. Also, Fig. 3 ▸(*b*) shows Δ*E*_T_ as a function of the X-ray energy with Si(220) and Si(311) DCCMs obtained from fitting the Fermi edge. Here, filled circles indicate the experimental data. Also, the dashed lines show the theoretical bandwidths of the X-rays from analyses with the DuMond diagrams considering the angular divergence of the incident beams. With increasing photon energy, Δ*E*_T_ and the theoretical bandwidths become larger. Similar tendencies of the total resolution as a function of X-ray energy with DCCMs have been reported previously (Yasui *et al.*, 2023 ▸). With the Si(311) DCCM, photoelectron measurements with a high-energy resolution of around 150–300 meV are achievable over the entire region. Although the energy resolution of the Si(220) DCCM is approximately twice as broad as that of the Si(311), it provides approximately four times greater X-ray flux, which is advantageous for flux-demanding applications. Therefore, efﬁcient measurements are possible by selecting the appropriate DCCM for individual applications.

### 3.2. Focused beam size and flux at sample

The focused beam size of the Wolter-type focusing mirrors in EH1 and EH2 was evaluated using a photodiode behind a 200 µm-diameter Au wire for a knife-edge scan. In the case of the excitation energy of 7.94 keV, the size of the focused beam at the sample position was measured to be 1.3 µm (V) × 23 µm (H) and 1.0 µm (V) × 13 µm (H) for EH1 and EH2, respectively. The high beam ﬂuxes of 6.0 × 10^12^ photons s^−1^ for EH1 and 4.6 × 10^12^ photons s^−1^ for EH2 were experimentally measured using the Si(311) DCCM at *h*ν = 7.94 keV. Furthermore, we conﬁrmed that this focusing performance can be reproduced once the mirror is retracted and re-installed. Also, by moving the mirror 50 mm in the downstream direction, the beam size for both mirrors can be enlarged to about 20 µm (V) × 50 µm (H). The beam density decreases by a factor of about 50 while maintaining the beam intensity. The beam profile changes upon defocusing, which occurs when the mirror is displaced. Near the focal point, the profile approximates a Gaussian distribution, whereas it gradually becomes a rectangular shape at positions further away. This is an effective technique to suppress the charge-up effect and X-ray irradiation damage for the insulating and soft materials.

## 4. Applications

### 4.1. High-throughput HAXPES system (EH1)

**Figure 4:**
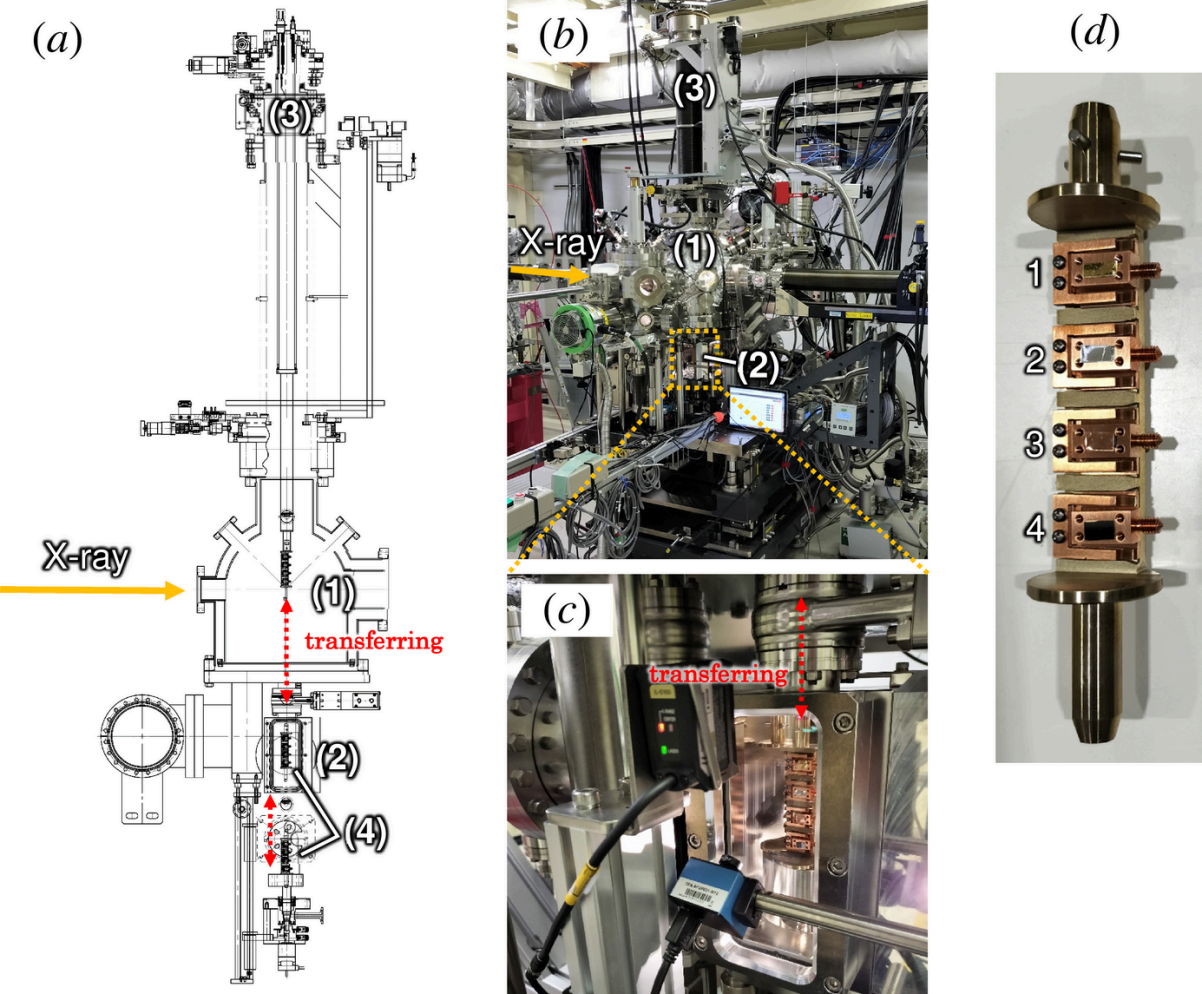
(*a*) Schematic and (*b*) photograph of the high-throughput HAXPES system comprising the (1) analysis chamber, (2) lower load lock and (3) six-axis manipulator. (*c*) Enlarged photograph of load-lock chamber. (*d*) Sample holder stocker.

For EH1, we have developed a high-throughput HAXPES system with automated measurement and a sample exchanger to meet the increasing demands in recent years. Figs. 4 ▸(*a*) and 4 ▸(*b*) show an overview of the system, which consists of the following components: (1) an analysis chamber, (2) a lower load lock, (3) a high-precision sample positioning mechanism (six-axis manipulator) and (4) a sample holder stocker. The analysis chamber (base pressure of ∼5 × 10^−6^ Pa) is equipped with a hemispherical photoelectron analyzer in the horizontal geometry. The lower load lock chamber, which has a base pressure of ∼1 × 10^−5^ Pa, is designed as an auxiliary chamber for quick exchange of the sample holder stocker and evacuation from ambient into UHV without disrupting the vacuum in the analysis chamber, as shown in Fig. 4 ▸(*c*). The sample holders with samples are set on the sample holder stocker shown in the photograph of Fig. 4 ▸(*d*). Note that a maximum of four sample holders can be set on the sample holder stocker. This sample holder is compatible not only with the ambient-pressure HAXPES system in EH2 but also with the two HAXPES systems in BL09XU. The sample holder stocker is set in the lower load-lock chamber, which can hold up to four sample holder stockers. To exchange the stocker, a turntable at the bottom of the chamber rotates to select the desired stocker. The end-effector of the six-axis manipulator is equipped with a locking mechanism designed to secure the three pins located on the top of the stocker. The six-axis manipulator approaches the sample holder stocker from the analysis chamber, then grabs and transfers it to the analysis chamber without breaking the vacuum, as indicated by the double-ended red arrow labeled ‘transferring’ in Figs. 4 ▸(*a*) and 4 ▸(*c*). This system allows for the simultaneous introduction and evacuation of multiple samples into the vacuum, thereby enabling efficient sample transport and measurements. This six-axis manipulator is not equipped with a heating or cooling system, as it is specifically designed for high-throughput measurements. In contrast, the side-load type manipulator shown in Fig. 2 ▸(*a*) enables *in situ* measurements via heating (up to 500°C) and voltage application to the sample. We have also introduced the *PEAK* analyzer control software developed by Scienta Omicron AB for the automated HAXPES measurement systems. We linked the Python based *PEAK* software to the instrument control platform BL-774, enabling integrated operations such as sample manipulation, exchanging and transferring the sample holder stocker, automatic position adjusting, and attenuator setting. Additionally, to improve usability, the command-line interface based on Python is encapsulated within the GUI based on a web API (Yasui *et al.*, 2024 ▸). This system is shared with the HAXPES systems at BL09XU and has several advantageous characteristics. For example, automated and continuous HAXPES measurements through multiple samples and measurement positions are available. In addition, fine alignment can be achieved by scanning the sample position to maximize the count rate of the intended energy range of the photoelectrons. After setting the measurement positions and conditions on the GUI, including the energy range, *E*_p_, energy step and dwell time, this system can automatically perform measurements at multiple positions. These features are quite advantageous when users want to execute the measurements of a number of samples at various experimental conditions, such as take-off angles, in a routine way. In some cases, continuous automated HAXPES measurements for several hours to around 10 h or even longer are possible. In addition, the design of the web API based GUI is simple and easy to operate even for inexperienced users.

### 4.2. Ambient-pressure HAXPES (EH2)

The ambient-pressure HAXPES system has been installed in EH2. Unlike conventional XPS instruments that require an ultrahigh-vacuum environment, the system employs a differential pumping chamber in front of the analyzer. In addition, a special photoelectron capture nozzle with a small aperture diameter is implemented to accommodate high gas pressure. This configuration enables XPS measurements under a gas atmosphere (Ogletree *et al.*, 2002 ▸). A Wolter-type mirror focuses the incident beam onto the sample in the chamber. The beam is focused to a size of 1.0 µm (V) × 13 µm (H) at the sample position. With a typical incidence angle of 3°, a footprint is elongated on the sample surface to approximately 1.0 µm (V) × 250 µm (H).

As an analyzer, a Scienta Omicron R4000 Hipp-2 analyzer is utilized, positioned at 90° to the beam in the horizontal plane. To optimize measurements under ambient pressure, a custom-made nozzle with a smaller-aperture diameter can be utilized instead of the standard 300 µm-diameter aperture (Takagi, Nakamura *et al.*, 2017 ▸). For example, a 30 µm-diameter aperture enables a shorter working distance, minimizing photoelectron scattering by the ambient gas and facilitating measurements at elevated gas pressures (Kahk *et al.*, 2015 ▸). Alternatively, an 80 µm (H) × 20 µm (V) rectangular aperture can be employed to efficiently collect photoelectrons and enhance signal intensity, which is particularly advantageous given the horizontally extended beam footprint on the sample. Consequently, the optimal aperture shape can be selected based on the specific experimental objectives.

Sample manipulation is achieved with a four-axis manipulator introduced from the top of the measurement chamber, enabling precise control of the measurement position. Various individual sample stages are available to accommodate different measurement requirements, including heating, voltage application and dip-and-pull method. For heating, a built-in ceramic heater allows the sample to be heated up to 500°C, facilitating *operando* measurements of reaction states at high temperatures. For voltage application, voltage can be applied and current passed through the sample via a feedthrough, with the sample insulated from the chamber ground. In the dip-and-pull method, three electrolyte electrodes (working, counter and reference electrodes) are provided and can be controlled using a potentiostat. Similarly, the lower chamber section incorporates an *xyz* stage and a rotation stage, facilitating measurements of fuel cells (Takagi *et al.*, 2018 ▸) and precise positioning of the electrolyte container for the dip-and-pull method (Oh *et al.*, 2024 ▸). Additionally, optical *operando* reaction measurements are possible by irradiating light onto the sample using a quartz based light guide introduced from the chamber port. Gas lines for H_2_, O_2_, and inert gases like He, N_2_ and Ar are installed for measurements under controlled gas atmospheres. An auxiliary gas line is also available for introducing user-supplied gases, such as CO_2_. Gas inflow to the chamber is precisely regulated by a mass-flow meter, while the exhaust volume is adjusted using a butterfly valve located upstream of the dry pump. A PID control loop incorporating a vacuum gauge maintains the desired gas pressure within the measurement chamber.

**Figure 5:**
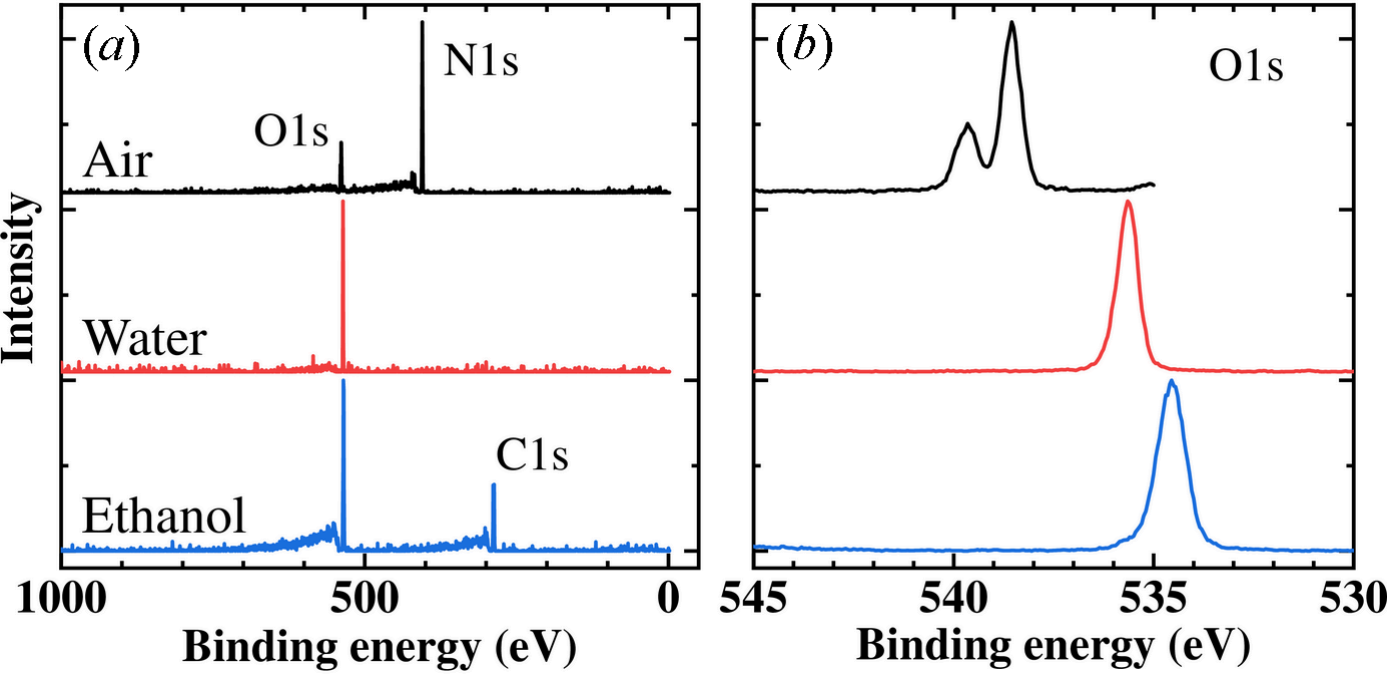
HAXPES spectra performed for air at 10 kPa, water vapor at 4 kPa and ethanol at 8 kPa. (*a*) Survey scans. (*b*) O 1*s* spectra. For survey scans, the per-point dwell and overall measurement times are 0.1 s and 5 min, respectively, and 0.6 s and 8 min for narrow scans. Each spectrum is normalized by the main peak intensity.

Fig. 5 ▸ presents the results of HAXPES measurements performed on gas molecules introduced into the measurement chamber. Measurements were conducted at 10 kPa air, 4 kPa water vapor (saturated vapor pressure) and 8 kPa ethanol using a 7.94 keV incident photon energy. The Si(311) crystal was chosen for the DCCM, and the analyzer transmission energy was set to 200 eV. The total energy resolution was estimated to be approximately 400 meV, based on measurements of the Fermi edge of Au foil. Each spectrum was normalized by peak intensity, and the difference in intensity between each was about a factor of two. Survey scans revealed the presence of oxygen and nitro­gen in the air sample, while only the oxygen peak was observed in the water vapor spectrum. In the ethanol spectrum, both oxygen and carbon peaks were detected. Narrow scans of the O 1*s* peak for each gas demonstrated distinct spectral features. The oxygen peak in the air spectrum exhibited spin–orbit splitting with a separation of 1.1 eV, characteristic of molecular oxygen (Hedman *et al.*, 1969 ▸). In contrast, water vapor displayed a single peak. The oxygen peak in the ethanol spectrum exhibited a chemical shift relative to that of water vapor. Furthermore, the C1*s* peak in the ethanol spectrum exhibited a clear chemical shift splitting, accurately reflecting the different chemical environments of the carbon atoms within the molecule (figure not shown). These results unequivocally demonstrate the capability of HAXPES for precise chemical state analysis of gas molecules.

**Figure 6:**
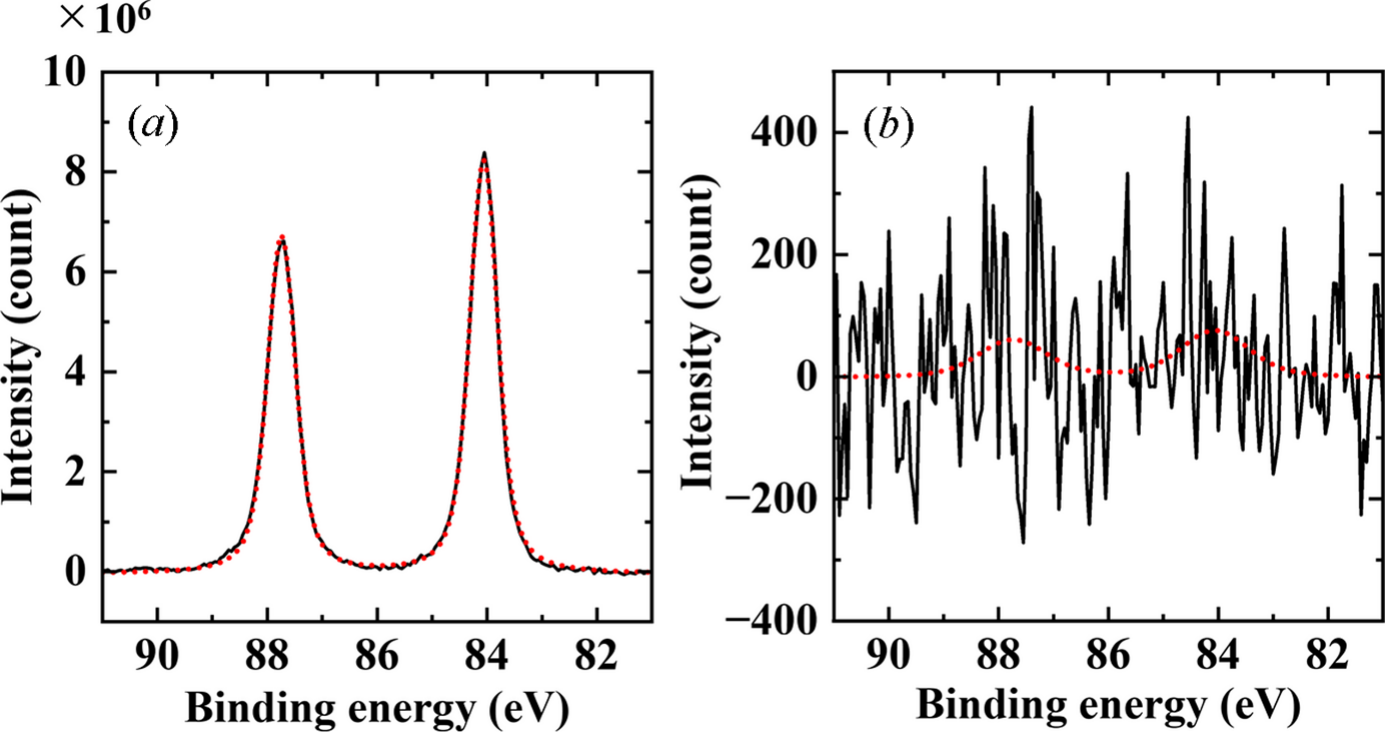
Au 4*f* HAXPES spectra under N_2_ gas at (*a*) 10 Pa and (*b*) 100000 Pa. The per-point dwell time was 0.2 s, with an overall measurement time of 2 min. The fitting results using the Voigt function are presented as a red dotted line.

Measurements of an Au film were performed under a nitro­gen gas atmosphere at two distinct pressures: 10 Pa (low vacuum) and 100000 Pa (atmospheric pressure). An 80 µm × 20 µm rectangular aperture was employed with a working distance of 60 µm. The results, presented in Fig. 6 ▸, indicate that the Au 4*f* peak is discernible through spectral fitting, despite a low signal-to-noise ratio at atmospheric pressure. The spectrum was acquired in 2 min, resulting in a poor signal-to-noise ratio. However, enhanced peak clarity could be achieved with extended measurement durations (Takagi, Nakamura *et al.*, 2017 ▸). As the measurement time was consistent across both conditions, the number of signal counts on the vertical axis represents the intensity ratio between low vacuum and atmospheric pressure. A comparative analysis of peak intensities reveals that the signal intensity at atmospheric pressure is 0.001% of that observed under low-vacuum conditions.

**Figure 7:**
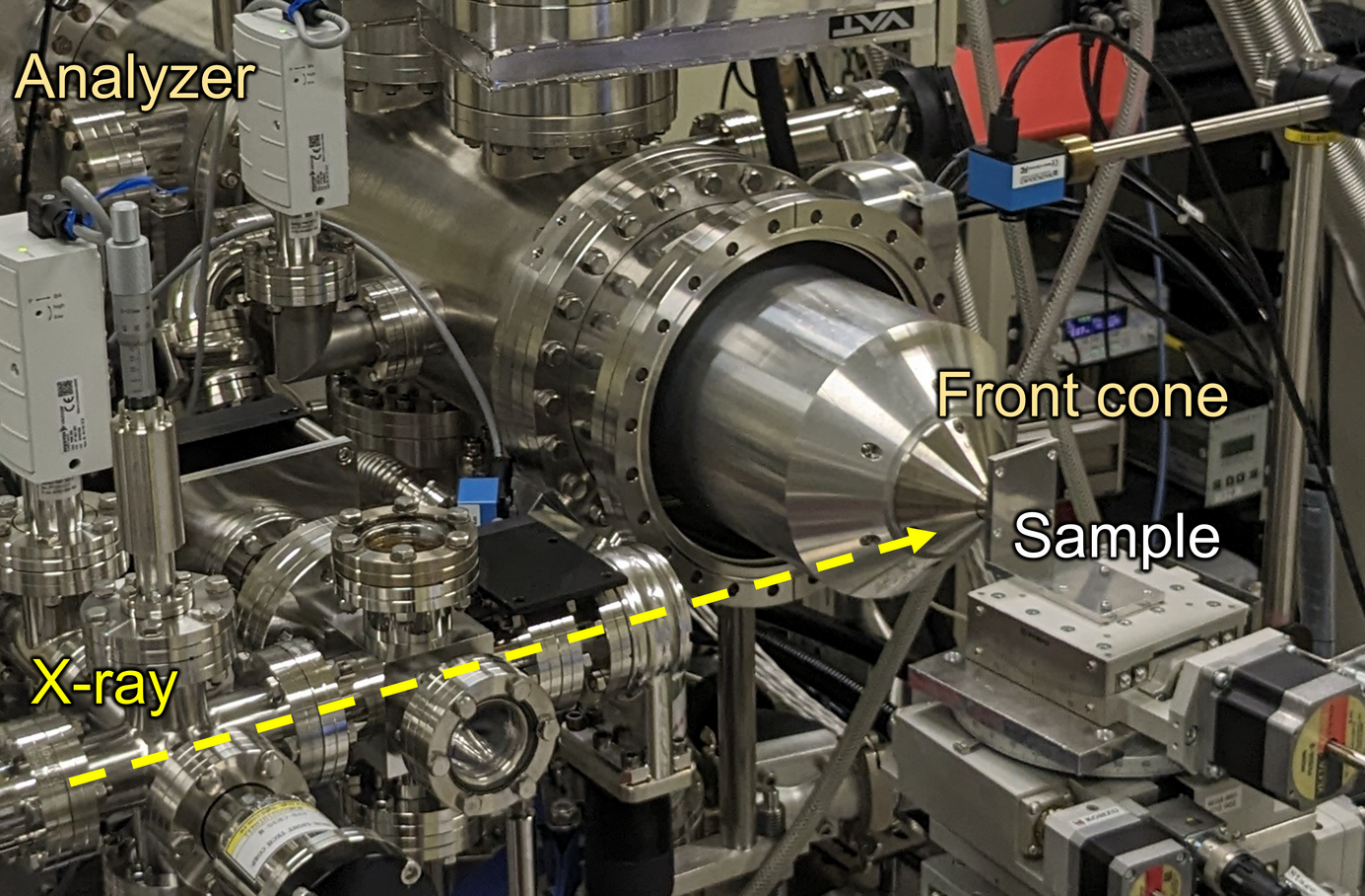
Photograph of HAXPES measurements under conditions where the measurement chamber has been removed.

The system’s ability to operate at atmospheric pressure eliminates the need for a measurement chamber to depressurize the sample environment. Consequently, measurements were conducted with the chamber removed, as shown in Fig. 7 ▸. This configuration allows for direct sample placement in front of the nozzle, significantly simplifying sample handling and enabling the analysis of large or contaminated samples that would traditionally be difficult to introduce into a measurement chamber.

**Figure 8:**
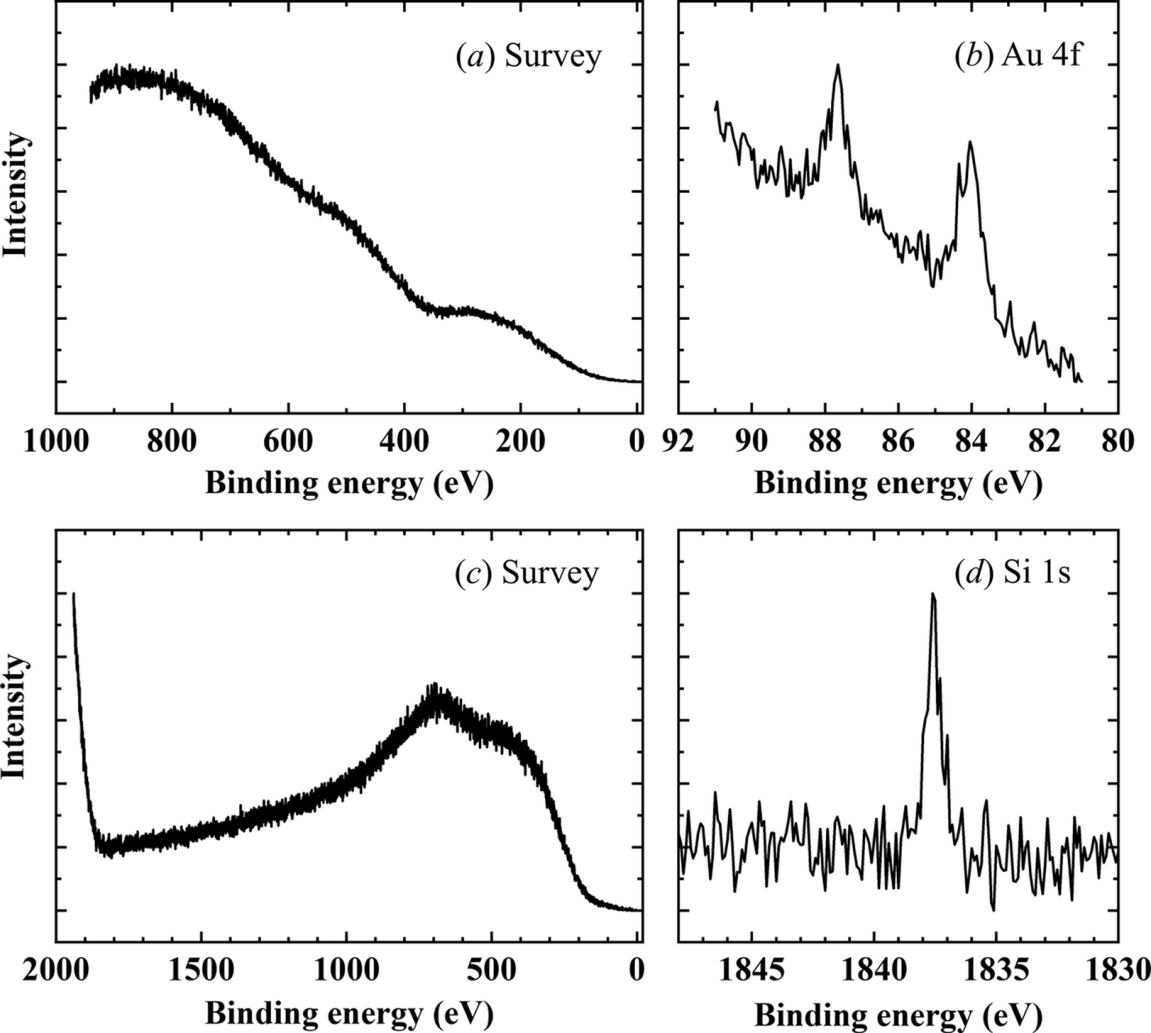
HAXPES measurements under atmospheric conditions. (*a*) Survey scan of Au thin film, (*b*) Au 4*f* peak of Au thin film, (*c*) survey scan of Si substrate and (*d*) Si 1*s* peak of Si substrate. For survey scans, the dwell time per point is 0.1 s. The total measurement times are 5 min for Au and 10 min for Si, attributed to variations in the measurement range. For narrow scans, the dwell time per point is 1 s for Au and 0.2 s for Si, resulting in total measurement times of 10 min for Au and 2 min for Si.

Fig. 8 ▸ presents the results of HAXPES measurements performed on an Au thin film and an Si substrate. A rectangular aperture 20 µm × 80 µm in size was employed, and the working distance was maintained at approximately 50 µm. Since the primary focus of this measurement was not solid–gas reactions at the surface, the working distance was minimized to reduce photoelectron scattering by the ambient gas. The spectra in Fig. 8 ▸(*a*) exhibit a significant background signal at higher binding energies relative to the inner-shell peaks. This background signal arises from photoelectrons that have lost energy through scattering interactions with the ambient gas. Despite this, weak spectral features were discernible. Accurate peak energy determination was achieved by carefully subtracting the background contribution. For the Au thin film, the Au 4*f* peak was successfully resolved [Fig. 8 ▸(*b*)]. Peak fitting analysis revealed a spin–orbit splitting of 3.7 eV between the Au 4*f*_7/2_ and Au 4*f*_5/2_ components, consistent with values reported in the literature. In the case of the Si substrate as shown in Figs. 8 ▸(*c*) and 8 ▸(*d*), the bulk sensitivity of HAXPES enabled the detection of the pure silicon peak beneath the native oxide layer (Takata *et al.*, 2005 ▸).

## 5. Summary

In this work, we have presented the design and performance evaluation of the beamline BL46XU at SPring-8 upgraded as a beamline dedicated to HAXPES techniques. To enable highly efficient and advanced HAXPES measurements, advanced optical instruments such as DCCM and Wolter-type focusing mirrors were installed and high photon flux and high energy resolution in the energy range from 4.9 keV to 21.8 keV were achieved. Also, the instrument control system BL-774 allows us to operate all instruments seamlessly. This upgrade will enable users to utilize the appropriate instruments in different ways according to the measurement target and analysis purpose, thus leading to further achievements and improved convenience for users. In particular, the development of the high-throughput HAXPES system in combination with BL-774 and *PEAK* has enabled highly efficient automated measurements, and the ambient-pressure HAXPES system is expected to be widely utilized in various research fields related to solid–gas and solid–liquid interfacial reactions.

## Figures and Tables

**Table 1:** HRM specifications

		Bragg angle (°)	Energy (keV)	Δ*E*_M_/*h*ν (×10^−5^)
DCCM	Si(220)	10–45	18.6–4.6	3.8
	Si(311)	10–45	21.8–5.4	1.6

**Table 2:** Optical parameters of Wolter-type focusing mirrors

	EH1	EH2
Focusing size (V × H) (µm)	0.6 × 23.0	0.3 × 13.0
Glancing angle (mrad)	5.0, 5.0	5.0, 5.0
Spatial acceptance (mm)	1.3 × 2.0	1.3 × 2.0
Working distance (m)	1.45	0.86
Magnification factor	1/29	1/48
Cut-off energy (keV)	∼12.0	∼12.0
Surface coating	Ru	Ru
